# Vascularized fibular flap and custom-made synthesis in post-traumatic ulnar diaphyseal pseudarthrosis: a case report

**DOI:** 10.1186/s13256-023-04108-4

**Published:** 2023-10-08

**Authors:** Filippo Zanotti, Gabriele Molteni, Umberto Lavagnolo, Riccardo Nocini, Massimo Corain

**Affiliations:** 1 Hand Surgery Unit, University Hospital of Verona, Verona, Italy; 2 Head and Neck Department, University Hospital of Verona, Verona, Italy

**Keywords:** Vascularized fibular graft, Custom-made, Pseudarthrosis, Ulnar nonunion, Case report

## Abstract

**Background:**

Although isolated fractures of the ulnar shaft are considered common and relatively benign injuries, numerous complications can arise especially in the context of suboptimal care pathways. For pediatric patients, however, there is no single indication of the surgical approach. In the context of the management of these complications, it is known that the vascularized fibular graft has numerous advantages and indications in the treatment of recurrent pseudarthrosis. However, in revision surgery the frequent occurrence of anatomical subversions requires the use of fixation means adapted to the individual patient. We present a clinical case of an adult patient suffering from post-traumatic ulnar pseudarthrosis treated with autologous vascularized fibula grafts and 3D-planned custom-made plate.

**Case presentation:**

A 38-year-old Ivorian woman came to our attention with a painful nonunion of the ulnar shaft and significant dysmorphism of the left forearm, with shortening and flexion of the limb as an outcome of unspecified road trauma in childhood. No alterations of the nerve compartment were reported. As far as detectable, she had undergone autologous bone grafting and implantation of questionable synthetic means, without acute treatment. Since we evaluated the patient (2012), we have performed two debridement surgeries, associated with autologous avascular bone graft from the iliac crest and plate fixation (2012 and 2014). In both cases, rupture of the fixation media was observed. In 2021, the pseudarthrosis was treated with a vascularized fibular bone graft. The subverted radius and ulna anatomy and poor bone quality required patient-specific reconstruction of the pseudarthrosic ulna from a 3D scan and the production of custom-made plate and screws, supported by the creation of special guides for drilling and by optimizing the positioning of screws with preoperative digital models. In the postoperative period, regular follow-up visits with X-rays evaluations were performed at 1, 3 and 6 months after surgery. No inflammatory reactions or local rejection were found. The fibula graft healed at the proximal ulnar junction six months after the operation while it took eight months to heal at the distal junction. Functionally, we observed a pain reduction and a range-of-motion preservation.

**Conclusions:**

The multiple failures of diaphyseal reconstruction with avascular bone grafts have forced the indication to the vascularized fibular flap. This case is a unique experience but we believe that the association between vascularized bone graft and the potential for customization through 3D planning represents a valid surgical potentiality in complex cases of post-traumatic reconstruction.

## Background

Whereas isolated ulnar shaft fractures are considered common injuries and deemed relatively benign lesions usually resulting from direct traumas to the ulna, they can also be afflicted by variable degrees of complications, such as nonunion, radioulnar synostosis and loss of motion [1]. There is still no agreement about the management of isolated ulnar shaft fractures [2] and the decision depends on fracture stability and surgeon preference, although ORIF is widely recommended in adults and in patients with unstable lesions. Pediatric patients, on the other hand, are often challenging in terms of ulnar fracture treatment. Recently, there is a growing inclination toward surgical management even in these cases, aiming for the most correct anatomic realignment [3–5, 14]. However, there is always the possibility of complications and this is especially true in pediatric patients who have received poor or wrong care. In the literature, the risk of nonunion or malunion occurs in less than 1% of fractures treated with closed reduction in children [6]. In adulthood, these outcomes can progress to nonunion, malformations and early joint degeneration with symptoms that can sometimes limit common daily activities [4].

We present a case of an adult patient with ulnar shaft atrophic nonunion after childhood trauma and a long history of upper limb discomfort. In particular, surgical planning was characterized by the association of a free autologous vascularized fibula flap with the implantation of a custom-made plate fixation device, adapted to the patient's subverted anatomy.

## Case report

**Fig. 1:**
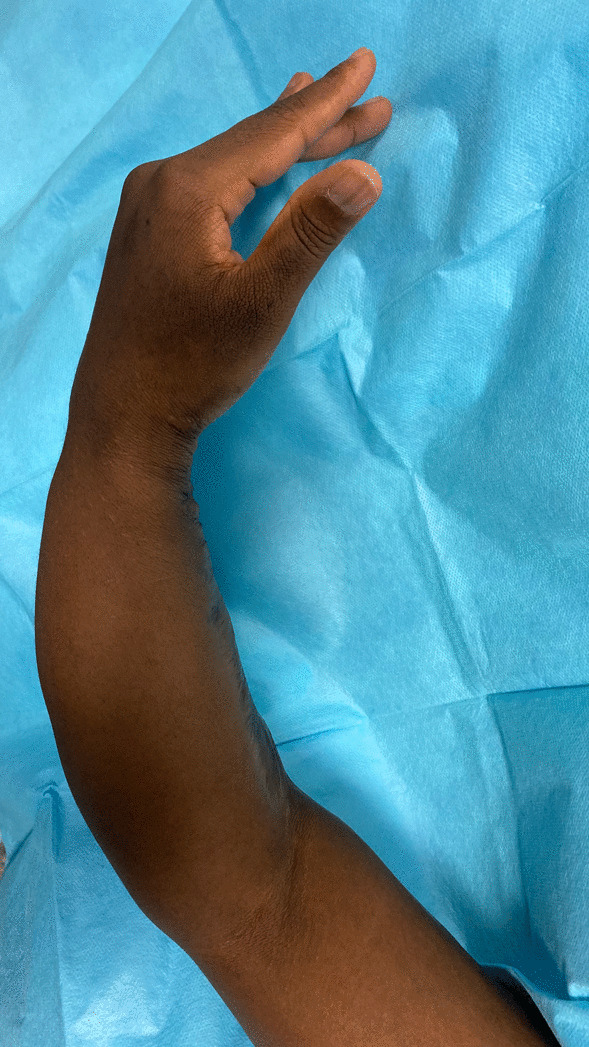
Forearm deformity previous surgery

A 38-year-old Ivorian woman came to our attention in September 2012 with a painful ulnar shaft nonunion and an important dysmorphism of her left forearm, with a shortening of 11 cm and varus deviation in comparison with the contralateral limb (Fig. 1). The clinical outcome was the result of an unspecified left upper forearm road trauma in childhood (approximatively at the age of 8 years). The patient arrived in Italy as an immigrant in 2012 (the correct date of arrival could not be determined due to a lack of documentation provided by the patient). During the examination, the patient was nulliparous, with a subsequent pregnancy in March 2014 (male child in good health), unemployed, and living with her partner. In her past medical history, she had type II diabetes mellitus since 2014 treated with metformin (1000 mg twice a day). She also had a history of retroperitoneal lymph node tuberculosis in 2015, treated for a year with a triple antibiotic regimen (isoniazid, rifampicin, ethambutol), with follow-up visits every 8 months by an infectious disease specialist. There was also a previous platelet disorder, likely of post-tubercular origin, which was being monitored by a hematologist and in a regressive stage. Additionally, she had cutaneous sarcoidosis with nodular-papular skin eruptions on her shins. The patient reported neither smoking nor habitual alcohol consumption. The patient’s vital signs were within normal limits (blood pressure: 130/85 mmHg; oxygen saturation 99% in ambient air; heart rate about 80 beats per minute; respiratory rate about 15 breaths per minute). The neurological physical examination did not report any neuropathic alterations in either the upper or lower limbs; all muscle groups were regularly recruited without significant deficits.

**Fig. 2:**
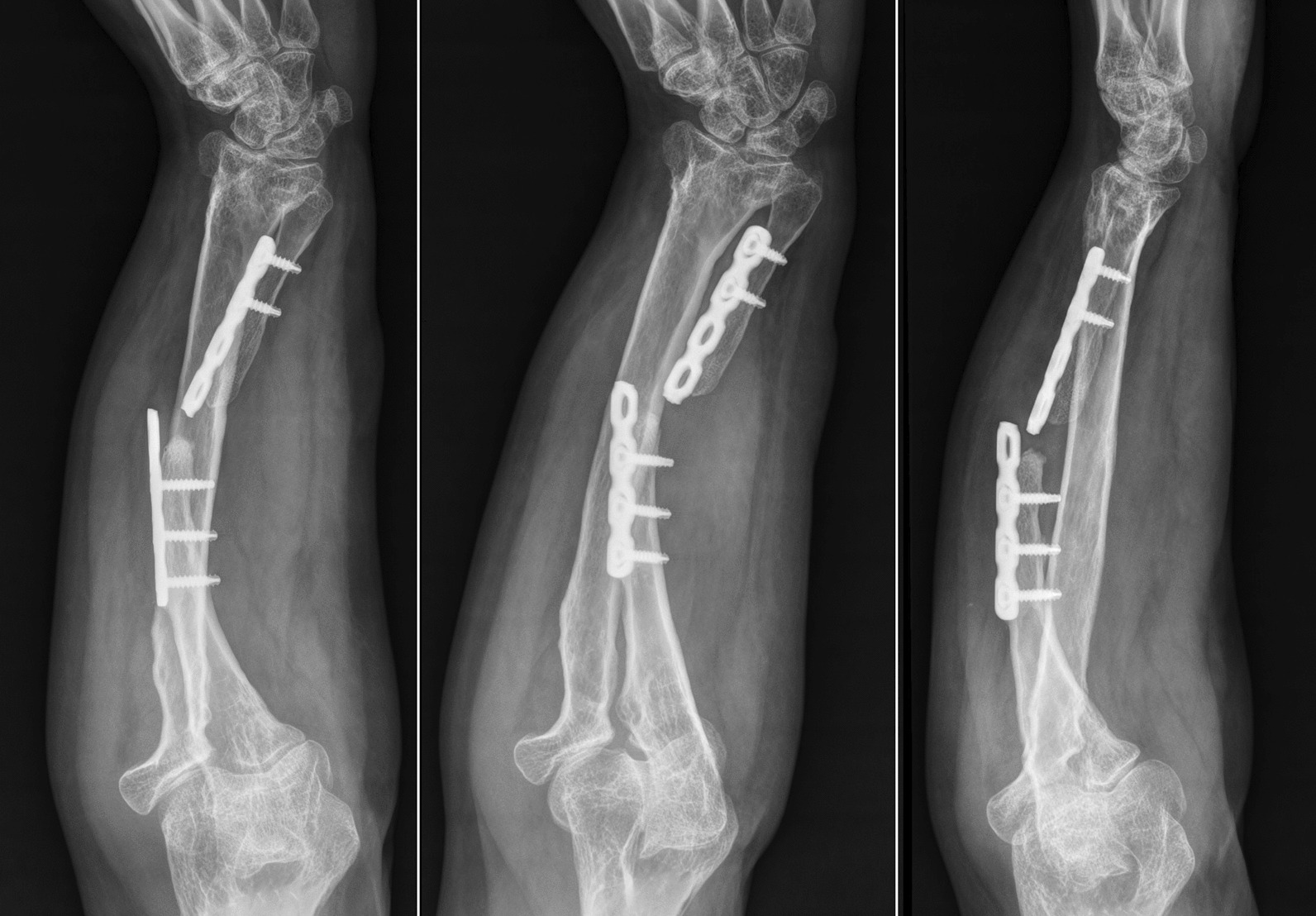
Last pre-op X-rays showing non union shaft site with evident bone graft loss and plate breakage

**Fig. 3:**
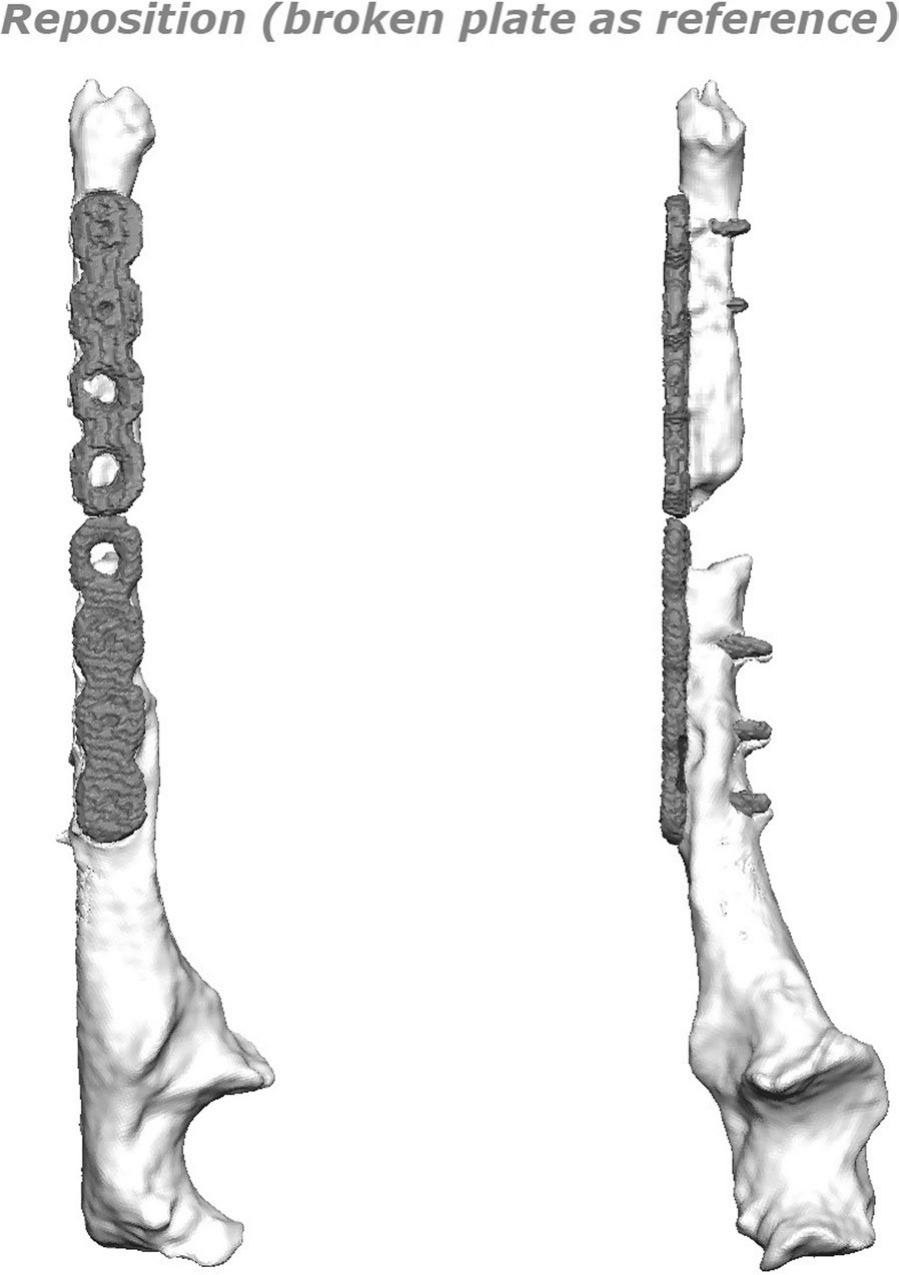
Use of the broken plate as a template to guide ulnar shaft reallinement

**Fig. 4:**
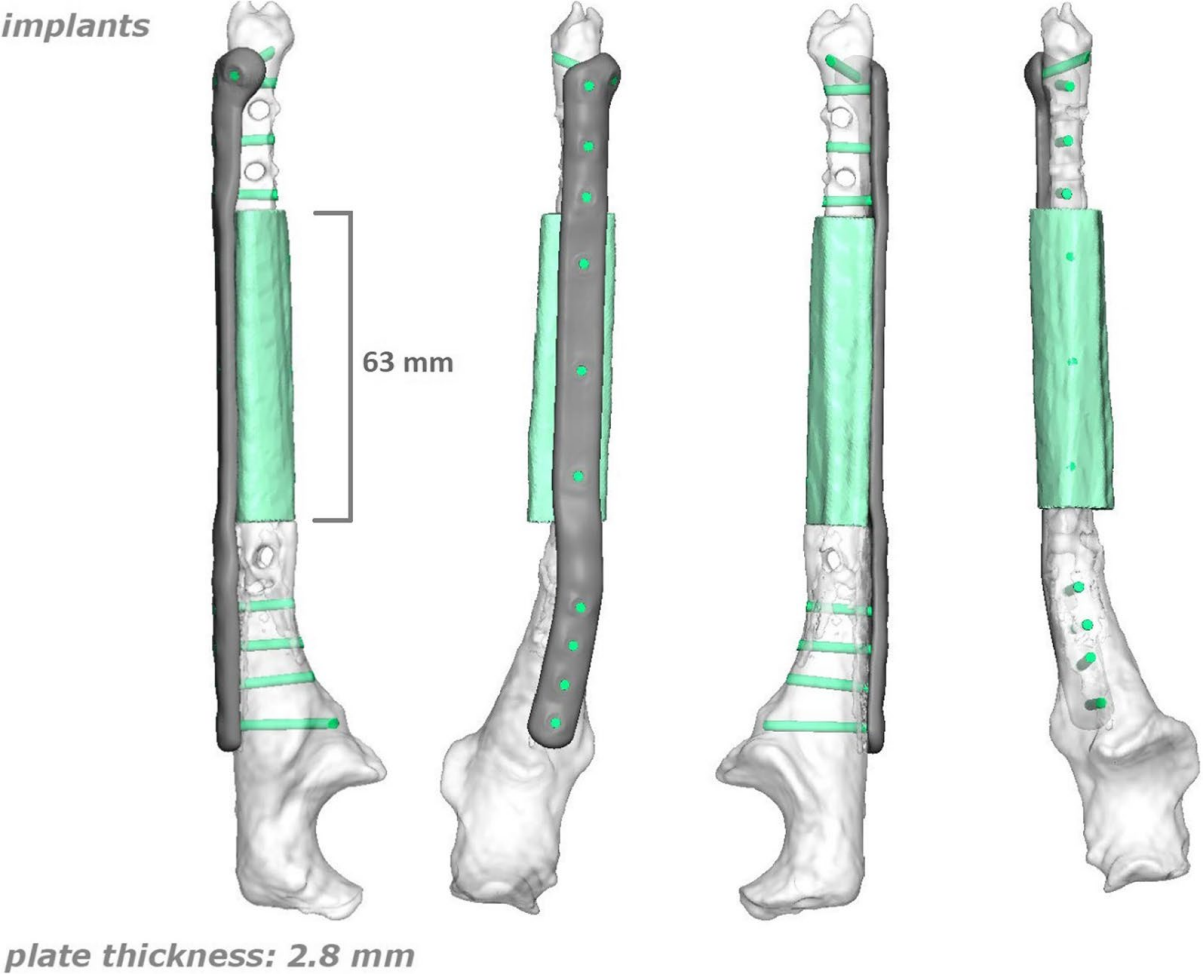
3D planning: note pre-existing screw holes (loci minoris resistentiae), accurate new screws number, lenght and positioning, amount of sclerotic bone extremities to resect at the nonunion site, precise fibula graft orientation and lenght needed to cover the resulting gap

**Fig. 5:**
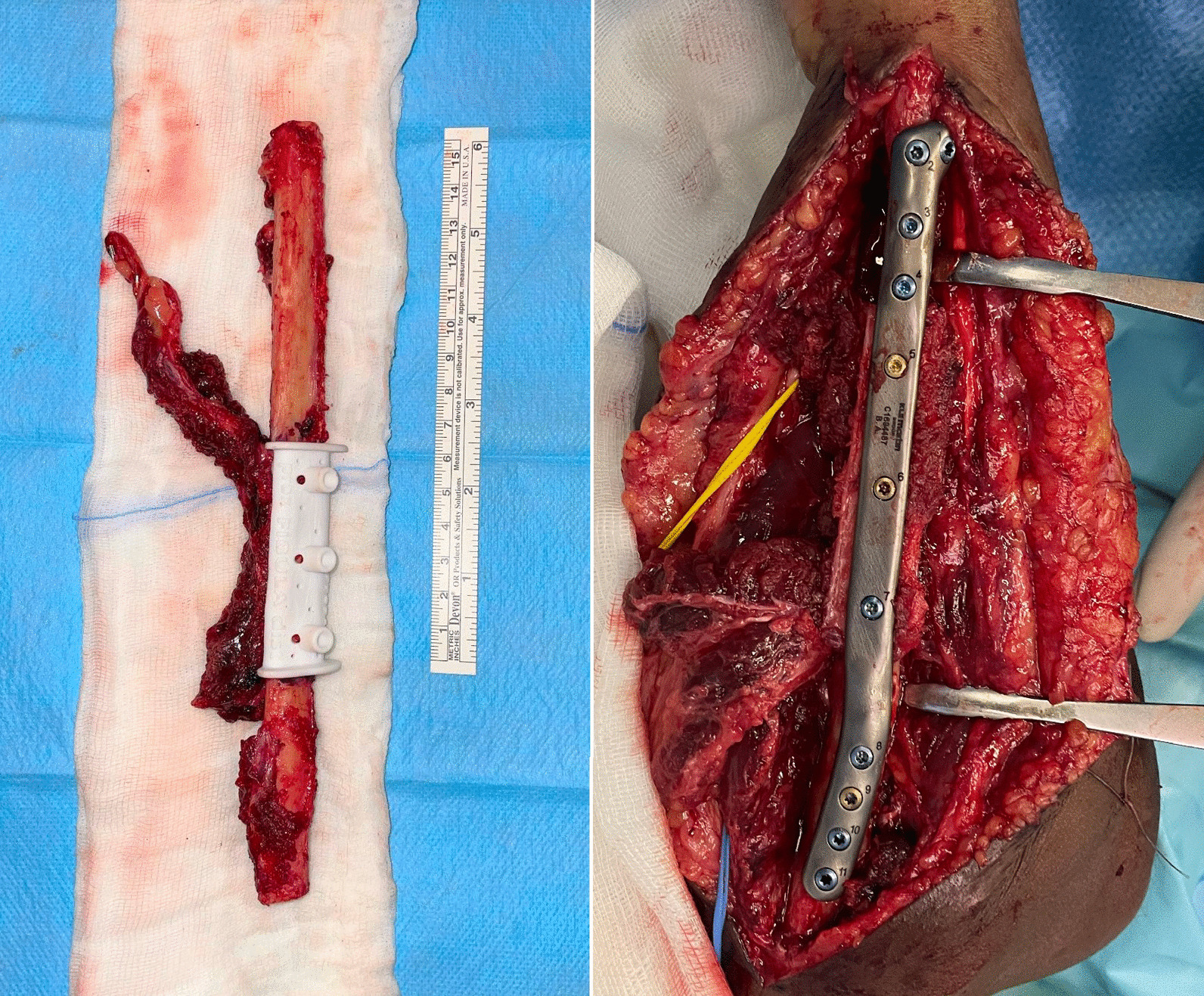
**a** Raised fibular flap with insetted cutting guide and vascular pedicle; **b** flap fixation into place after revascularization

**Fig. 6:**
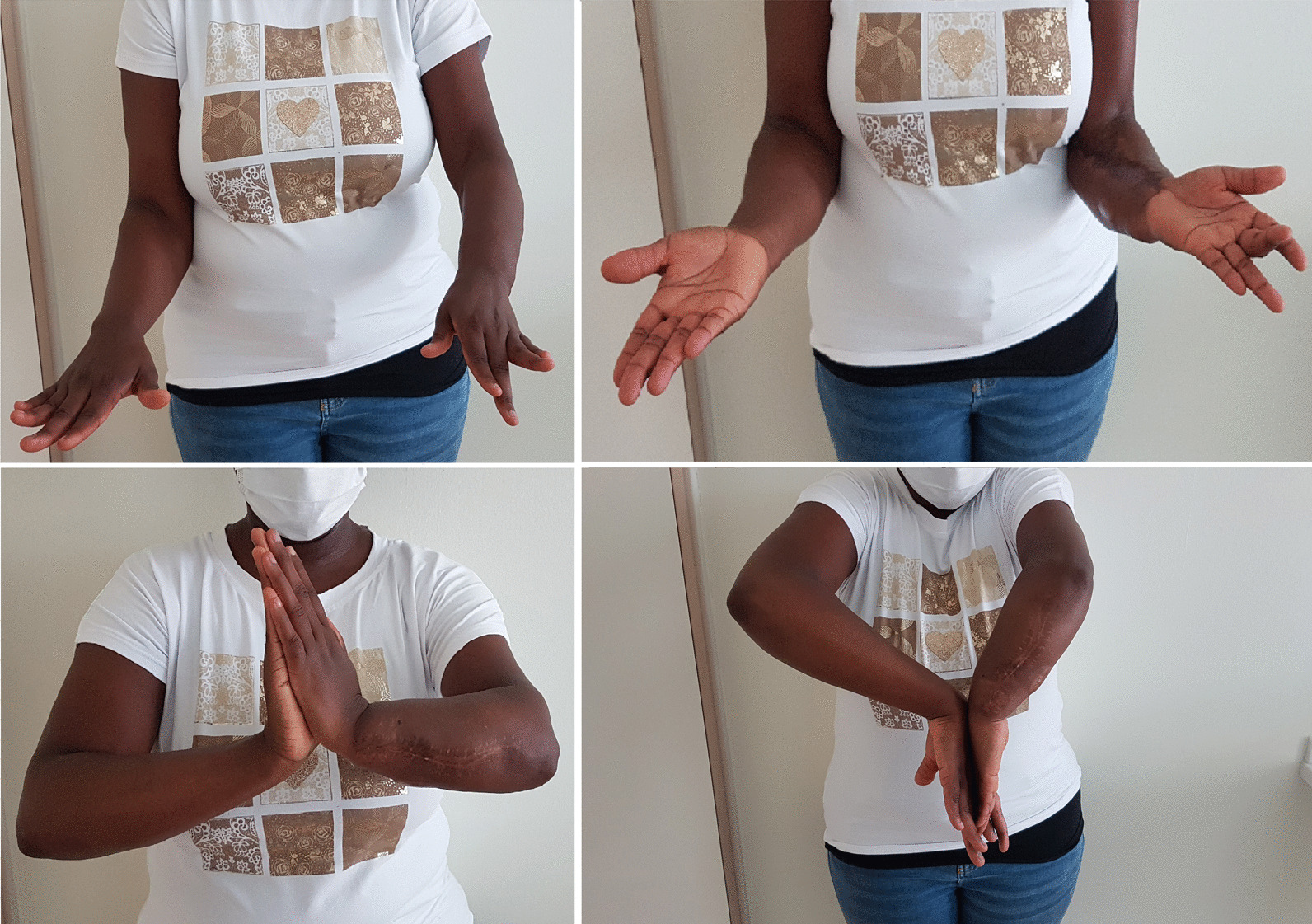
Clinical outcome. The patient had the possibility to get back to work

**Fig. 7:**
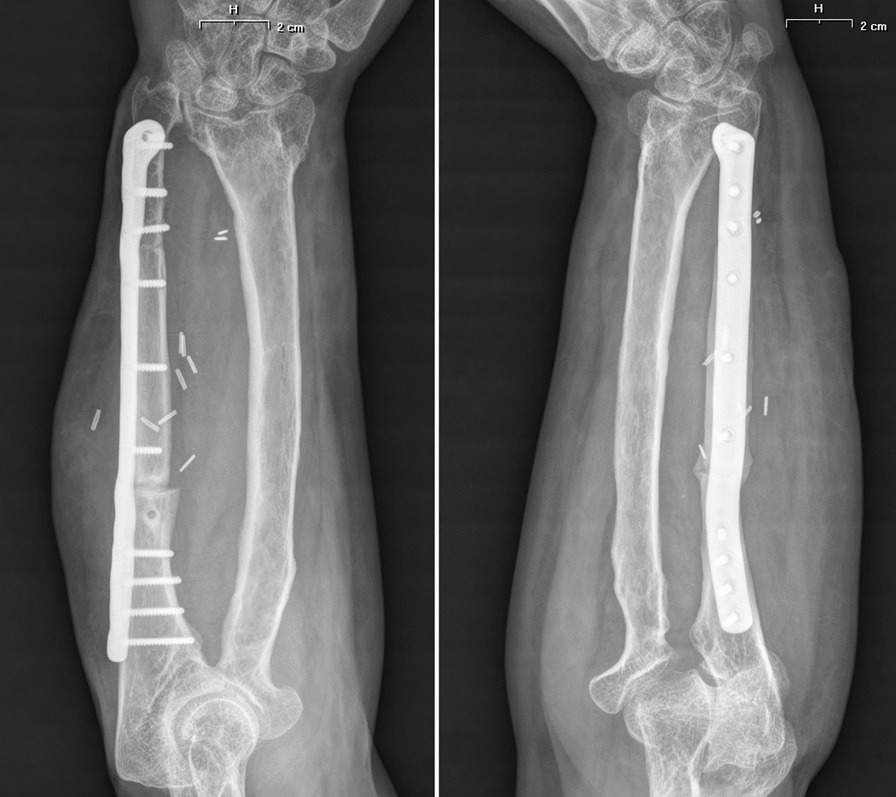
Follow-up X-rays at 8 months

As regards the orthopedic pathology, she did not receive any kind of acute treatment, followed by a subsequent undocumented surgical procedure with avascular autologous bone grafting. At the same time, an almost impossible to understand inner fixation device had been placed, of which there were no signs in the available X-rays. Her left wrist showed a significant range-of-motion reduction, about 60% than contralateral: limitation in pronation + 55°, in supination − 70°, in extension + 80°, in flexion − 30°), a long history of forearm pain (NRS 7), weakness and discomfort. However, DRUJ and elbow joints did not show major impairments, and there were no signs of peripheral nerves disfunction. She received two surgical attempts with debridement (September 2012 and August 2014), autologous avascular bone graft from iliac crest and plating fixation, resulted in poor outcome (nonunion with plate breakage) (Fig. 2). In March 2021 we decided to treat the nonunion with a vascularized free fibular flap. It is well established that this type of flap has numerous advantages and indications in the treatment of recurrent nonunion: matching anatomic shape compared to forearm long bones in terms of length and diameter, low risk of complications at the donor site, possibility of one-stage and multi-tissue reconstructions, high resistance to infections and strong alternative to avascular bone grafts [7–9]. We had another major issue to overcome, represented by the completely altered ulna and radius anatomy, which hindered the possibility to use a standard locking compression plate. One feasible and promising solution was to integrate in our planning the use of a patient-specific 3D custom made plate [10, 11]. Therefore, we decided to cooperate with KLS Martin to plan a patient-specific reconstruction of the atrophic ulna nonunion starting directly from a 3D scan of the affected limb and using the broken plate as a template to guide ulnar shaft realignment (Fig. 3). Accurate planning focused also on pre-existing screw holes (to be considered as *loci minoris resistentiae*), poor native bone quality and sclerotic extremities to resect at the nonunion site. The study resulted in a unique 2.8 mm width custom plate with a proper screw number and length for every section (Fig. 4), relative left ulna and right fibula cutting guides. In particular, the fibular flap length was carefully predetermined, in order to be 63 mm. The patient performed also a lower limbs CT angiography to assess fibula vascularization (in order to avoid anatomical variations in its course, that is peronea arteria magna), and a Doppler ultrasonography of the left forearm. Both of these exams ruled out possible complications. Surgery was performed in general anesthesia, by two teams of two surgeons each. Preoperatively antibiotic prophylaxis by administering Cefazolin 2 g intravenously and postoperatively crystalloid infusion therapy for volume replenishment was performed. In particular, no post-operative antibiotic prophylaxis was performed. The examinations performed in the preparatory and postoperative periods are shown in Table 1. At surgery, three biopsy samples of the preudoarthrosis focus were collected for microbiological studies, which were negative for the presence of pathogens. After free fibular flap elevation and ulnar shaft preparation, the flap was properly cut, inserted and stabilized with custom plate and screws. The entire surgical procedure was conducted under fluoroscopy guidance. Peroneal artery was anastomized in end-to-lateral configuration on the ulnar artery, while concomitant peroneal vein was anastomized in end-to-end configuration on a local tributary vein (Fig. 5). We didn’t modify the pre-existing ulna plus deformity, which could have been easy to perform due to ulnar shaft cut. This indication was considered after the relatively good pronation-supination movement showed before surgery by the patient and absence of ulnar wrist pain (Fig. 6). The forearm was then protected with a dorsal elbow splint for 4 weeks. In the post-operative course there were no anemias such as to require transfusion support. No complications were detected during surgical wound healing at the donor and recipient sites. We planned regular follow-up evaluations with X-rays at one, three and six months after surgery. Fibular flap healed at the proximal ulnar junction 6 months after operation, while it took up to eight months to appreciate healing in the distal junction point, probably due to scarce distal ulnar bone quality (Fig. 7). After removing the splint, physiotherapy and functional recovery sessions were performed for 4 months, in order to stimulate the patient to regain competence in pronation-supination and flexion–extension movements. After 6 months, we clinically assessed functional improvement with significant pain reduction (NRS 2) and a range-of-motion restoration as it was before the surgery.

**Table 1:** Pre- and postoperative blood tests. No urine tests were performed

	Preoperative	Postoperative	
Hemoglobin	13.3	10.6	g/dl
Erythrocytes	4.85	3.84	10^12^/l
Hematocrit	41	34	%
Platelets	174	132	10^9^/l
Leukocytes	5.41	6.53	10^9^/l
PT	1.07	1.01	INR
APTT	1.17	1.12	ratio
Fibrinogen	3.5	3.1	g/l
Urea	25.2	32.4	mg/dl
Creatinine	0.76	0.98	mg/dl
Sodium	139	142	mmol/l
Potassium	3.66	3.61	mmol/l
Chlorine	101	103	mmol/l
Glucose	71	87	mg/dl

*PT* prothrombin time,* APTT* activated partial thromboplastin time

## Discussion and conclusions

The case report discusses the successful use of a vascularized free fibular flap in upper limb post-traumatic reconstruction, which is a well-established practice. What makes this case unique compared to existing literature is the utilization of a patient-specific approach through 3D planning and customized plates. The patient had multiple failures in attempting diaphyseal reconstruction using avascular bone grafts, which led to the necessity of a vascularized bone flap. To address the complex anatomy and bone deformity, a CT scan of the affected limb was used to create an accurate 3D bone rendering. KLS Martin’s IPS® algorithm was employed to correct the acquired deformity and design a new custom titanium plate and cutting guides. These guides reduced the margin of error in calculations, determined the necessary flap length, and facilitated precise resection of atrophic nonunion areas. Additionally, the custom plate design accounted for the patient’s unique ulnar anatomy and poor bone quality, resulting in a construct with converging screws to bypass previous holes.

**Fig. 8:**
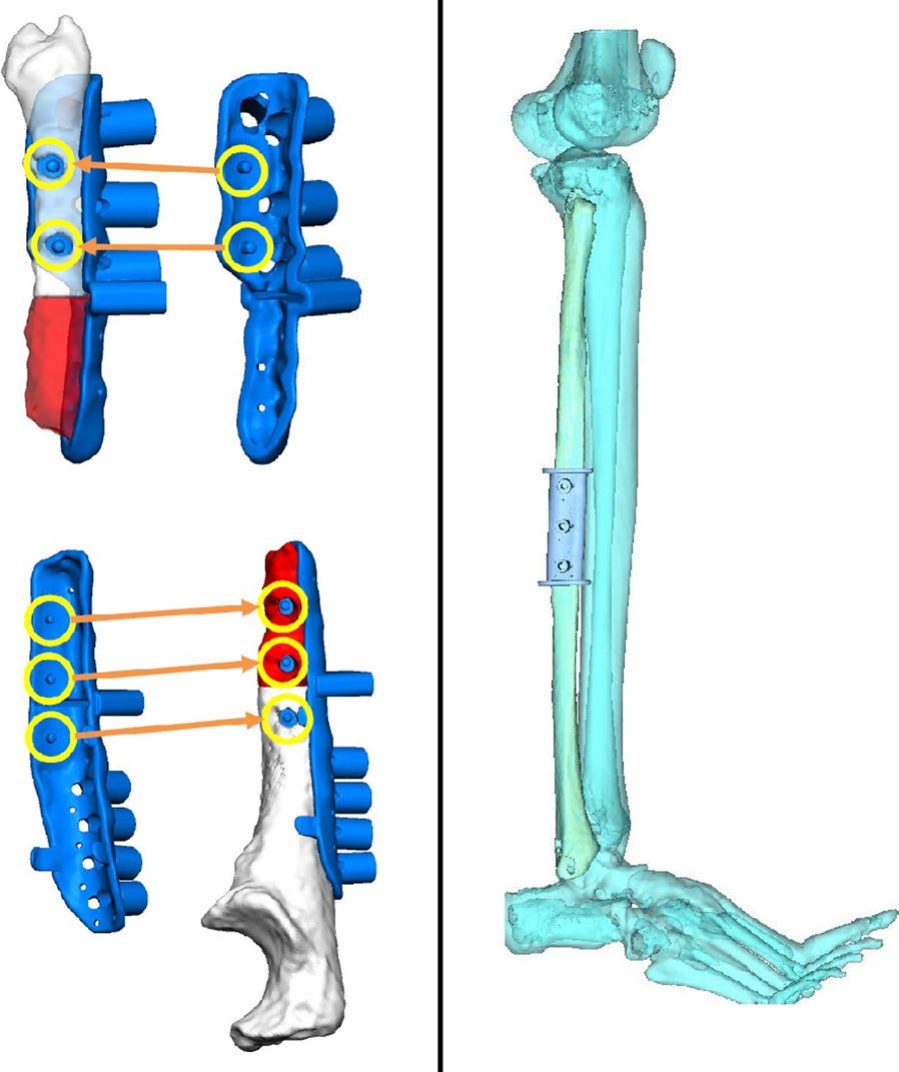
**a** Yellow circles indicate previous ulnar screw holes converted in reference points for precise guides placement; **b** 3D rendering showing fibular cutting guide

The use of a vascularized free fibular flap in upper limb post-traumatic reconstructions is a well-established practice with solid scientific foundations [12, 13]. In particular, the indication for its use in this patient was mandatory, considering multiple failures in attempting diaphyseal reconstruction using avascular bone grafts. Furthermore, the possibility of using customized plates is also well established in the reconstructive surgery of the upper limb [10, 11]. Through KLS Martin and their IPS® (Individual Patient Solutions) algorithm starting from a CT scan acquisition of the involved limb, it was possible to obtain an accurate 3D bone rendering and correct its acquired deformity to understand ulnar shaft shape as it was before plate breakage. Due to the anatomic discrepancies between left and right forearms and the impossibility to use the right side as a guide, this step was fundamental to be able to design a new custom plate (in titanium alloy Ti6Al4V, ASTM F136-02, (ELI grade 23), thickness 2.8 mm SLM (selective laser melting)) and appropriate cutting guides for both ulnar and fibular reference points. Guides have been essential to significantly reduce the margin of error in calculating the required fibular flap length, to precisely delimit bone areas to be resected at the level of atrophic nonunion and to pre-drill screw holes in correct number and position. In this specific case, preoperative planning using a 3D model made it possible to take into account previous screw holes and to convert them in reference points for correct guides housing, then to calculate the number of necessary screws and their type and length (both theoretical and recommended) (Fig. 8). Custom plate design took into account patient’s unique ulnar anatomy and in particular the poor ulnar bone quality in the distal diaphyseal fragment, using a construct with converging screws to bypass previous holes, especially near the ulna head. Furthermore, correct preoperative planning and the possibility of using cutting guides reduced surgical times and the use of intraoperative fluoroscopy, with less exposure for patient and operators. The clinical outcome was good with healing of the skin wounds and good bone consolidation of the flap. The detected function was equally positive and satisfactory for the patient, no longer limited in daily activities. It is important to underline that the surgery performed was not aimed at increasing range of motion, but rather at improving the painful component.

Probably in this patient it would have been possible to limit the malformation and the clinical consequences of the pseudarthrosis with an adequate early surgical treatment. In particular, the limited availability of access to adequate care in acute and the unclear medical history have complicated the clinical management of the patient.

In conclusion, we believe that the association between vascularized bone graft, patient-personalization power through 3D planning and dedicated devices represents a solid surgical weapon in complex cases of post-traumatic reconstruction in the presence of deformation of the upper limbs.

## Data Availability

All the patient’s clinical documentation is available in the medical records archived in the archive of the University Hospital of Verona, Italy.

## Abbreviations

ORIF: Open reduction internal fixation
DRUJ: Distal radial ulnar joint
NRS: Numeric Rate Scale
CT: Computed tomography
